# Protein regulator of cytokinesis 1: a potential oncogenic driver

**DOI:** 10.1186/s12943-023-01802-1

**Published:** 2023-08-10

**Authors:** Sijing Li, Omar Motiño, Flavia Lambertucci, Isabelle Martins, Li Sun, Guido Kroemer

**Affiliations:** 1 Centre de Recherche des Cordeliers, Université Paris Cité, Sorbonne Université, Equipe labellisée par la Ligue contre le cancer, Inserm U1138, Paris, France; 2 Metabolomics and Cell Biology Platforms, Gustave Roussy, Villejuif, France; 3 Faculté de Médecine, Université de Paris Saclay, Kremlin Bicêtre, France; 4 Institut du Cancer Paris CARPEM, Department of Biology, Hôpital Européen Georges Pompidou, Paris, HP France; 5 Jiangsu Key Laboratory of Drug Screening, China Pharmaceutical University, Nanjing, China

**Keywords:** Protein regulator of cytokinesis 1 (PRC1), Overexpression of PRC1, Prognostic clinical value, Functional roles of PRC1, Upstream regulators of PRC1, Th2 cells, Immune checkpoints

## Abstract

Protein regulator of cytokinesis 1 (PRC1) is involved in cytokinesis. Growing evidence suggests the association of PRC1 with multiple cancers. Here, we unveil that, in 28 cancer types, PRC1 is higher expressed in tumor tissues than in non-malignant tissues. Overexpression of PRC1 indicates unfavorable prognostic value, especially in ACC, LGG, KIRP, LICH, LUAD, MESO, PAAD, SARC and UCEC, while methylation of the PRC1 gene at sites associated with its inactivation has a favorable prognostic value in ACC, KIRP, LUAD, MESO, KIRP and LGG. Differentially expressed genes (DEGs) associated with high (> median) PRC1 expression contribute to key signaling pathways related with cell cycle, DNA damage and repair, EMT, cell migration, invasion and cell proliferation in most cancer types. More specifically, the DEGs involved in RAS/RAF/MAPK, PI3K/AKT, WNT, NOTCH, TGF-β, integrin, EMT process, focal adhesion, RHO GTPase-related pathway or microtubule cytoskeleton regulation are upregulated when PRC1 expression is above median, as confirmed for most cancers. Most importantly, high expression of PRC1 appears to be associated with an overabundance of poor-prognosis TH2 cells. Furthermore, positive correlations of PRC1 and some immune checkpoint genes (CD274, CTLA4, HAVCR2, LAG3, PDCD1, PDCD1LG2, TIGIT, and CD86) were observed in several cancers, especially BLCA, BRCA, KIRC, LUAD, LIHC, PRAD and THCA. These findings plead in favor of further studies validating the diagnostic and prognostic impact of PRC1 as well as the elaboration of pharmacological strategies for targeting PRC1.

**Supplementary Information:**

The online version contains supplementary material available at 10.1186/s12943-023-01802-1.

## Introduction

Protein regulator of cytokinesis 1, encoded by *PRC1*, is critical for cytokinesis (https://www.ncbi.nlm.nih.gov/refseq/). PRC1 is widely located in nucleus, cytoplasm, cytoskeleton, extracellular space, and plasma membrane, based on the information from COMPARTMENTS (https://compartments.jensenlab.org/Search), a subcellular localization database. Among the 19 transcripts of PRC1 (Table S1 and Figure S1), two transcripts (ENST00000556972.6 andENST00000394249.7) are the most abundant ones in normal tissues (Figure S2). In addition, PRC1 is specifically enhanced in testis, but expressed at low or undetectable levels in other normal human tissues (Figure S2). scRNA-seq data from HPA (The Human Protein Atlas) reveal that PRC1 is highly expressed in an array of distinct cell types across different human tissues (Figure S3).

Early in 2007, the oncogenic role of PRC1 has been reported in bladder cancer [1]. Since then a growing but limited number of studies have addressed the involvement of PRC1 in another 11 cancer types, including breast cancer, liver hepatocellular carcinoma (LIHC), prostate cancer (PCa), lung adenocarcinoma (LUAD), gastric cancer, colon cancer, ovarian cancer, Ewing sarcoma, esophageal cancer, oral squamous cell carcinoma (OSCC), liposarcoma, and nasopharyngeal carcinoma [2–12]. Despite the pivotal roles of PRC1 in cell cycle, cell proliferation, self-renewal, stemness, tumor growth, EMT (epithelial mesenchymal transition), migration, invasion, stemness, and metastasis in certain cancers, the underlying molecular mechanisms are not well defined except for a positive feedback loop between PRC1 and Wnt signaling [3, 5, 6, 9–11]. In addition, the upstream regulation of PRC1 remains poorly elucidated, though several transcription factors (EWSR1-FLI1, p53) and microRNAs (miR-194 and miR-143) have been shown to affect PRC1 expression [9, 11–13]. PRC1 has been reported to contribute to the immunosuppressive microenvironment of LIHC [14], suggesting that its potential immune effects should be investigated. Based on this fragmentary evidence, we embarked in an exhaustive bioinformatic characterization (for a detailed description see Supplemental text file 1) of PRC1 in human cancer.

## Results

### High expression and prognostic value of PRC1 in cancer

**Fig. 1:**
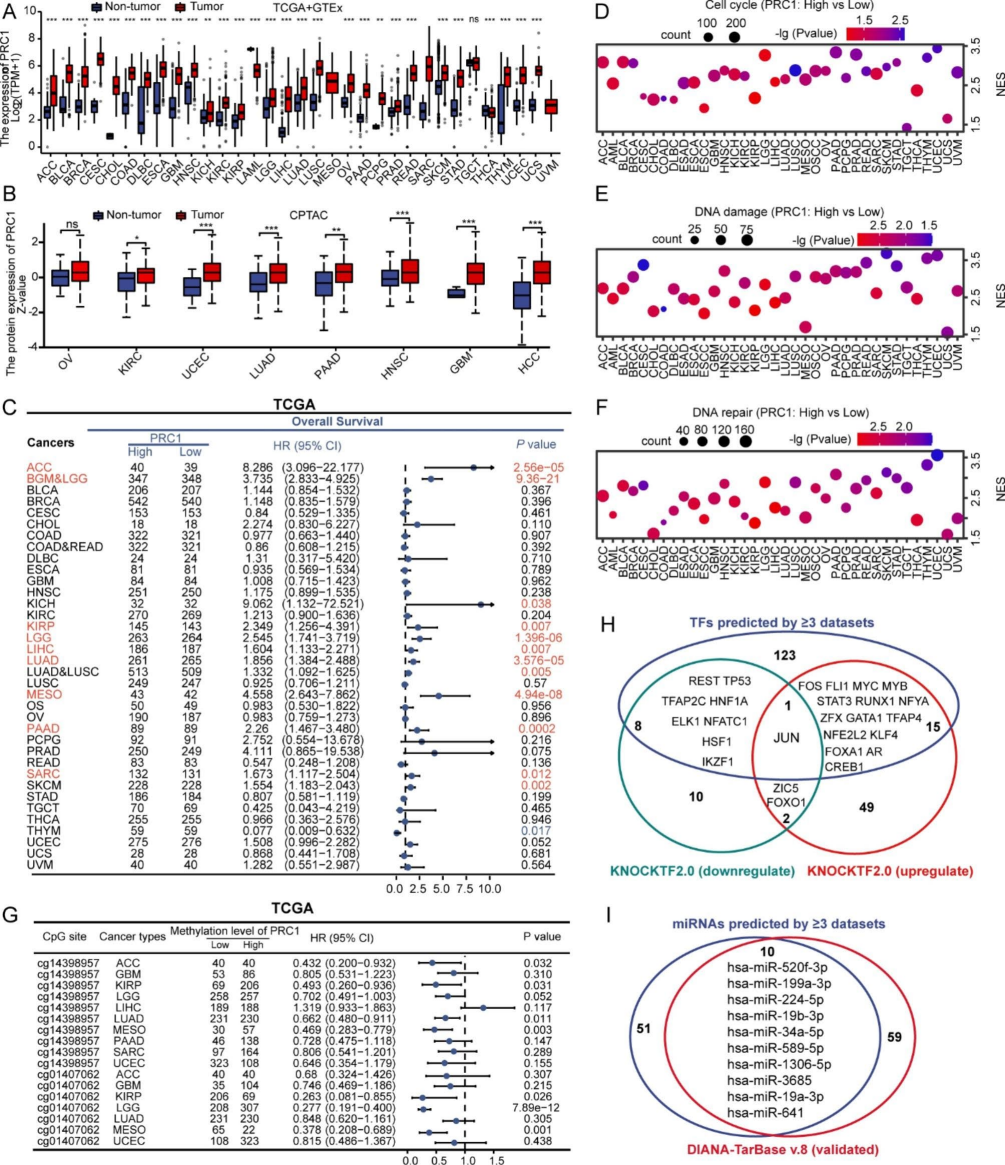
Clinical significance, functional roles and upstream regulators of PRC1. (**A**) The Expression profiles of PRC1 mRNA in cancers by the analysis of RNA-seq data from TCGA + GTEx database. (**B**) Differential expression of PRC1 protein in certain cancers according to the CPTAC database. (**C**) Analysis of overall survival (OS) based on PRC1 expression, with patients falling into PRC1^high^ (> median) and PRC1^low^ (< median) groups in each cancer type. GSEA-based analysis of cell cycle-related pathways (**D**), DNA damage (**E**) and DNA repair (**F**) were performed through analyzing PRC1-associated differential gene expression data in 36 cancer types. (**G**) Favorable prognostic value of PRC1 methylation in cancers determined using the MethSurv database. (**H**) Potential transcription factors responsible for regulating PRC1 expression. (**I**) Potential miRNAs targeting PRC1. Significance is indicated as follows: ns, p ≥ 0.05, *p < 0.05, **p < 0.01, and ***p < 0.001

RNA-seq data from TCGA/TCGA + GTEx datasets indicate that PRC1 mRNA is significantly higher in the majority of solid tumors compared with normal tissues except TGCT and LAML (Figure S4A, Fig. 1A). Paired sample analyses confirmed the elevation of PRC1 mRNA in tumor tissues compared with normal tissues for 17 cancers (Figure S4B). In addition, proteomic data from the CPTAC database corroborated high PRC1 expression in tumors compared with normal tissues in several cancers, including KIRC, UCEC, LUAD, PAAD, HNSC, GBM and HCC (Fig. 1B).

The prognostic value of PRC1, characterized by disease-specific survival (DSS), overall survival (OS), and progression-free interval (PFI), was initially assessed in 33 cancer types listed in TCGA. The results indicate that PRC1 might function as a prognostic biomarker in 8 cancers, including ACC, GBM&LGG, KIRP, LIHC, LUAD, MESO, PAAD and SARC, since high expression of PRC1 predicted poor DSS, OS and PFI (Figure S5, Fig. 1C, Figure S6). Consistently, KM-plotter analysis revealed that high expression of PRC1 is accompanied by short overall survival and relapse-free survival in KIRP, LIHC, LUAD, PAAD, SARC and UCEC (Figure S7A-B). In addition, time-dependent receiver operating characteristic (ROC) curves confirmed the prognostic value of PRC1 for OS, DSS and PFI in ACC, GBM&LGG, KICH, KIRP, LIHC, LUAD, MESO, PAAD, PCPG, PRAD and UCEC (Figure S8A-C).

### Functional role of PRC1 in malignancy

At single-cell (sc) RNAseq resolution, PRC1 was significantly and positively related to cell cycle, DNA damage, DNA repair, EMT process, cell invasion and proliferation in multiple cancer types (Figure S9). Based on bulk RNA-seq data from TCGA datasets, differential gene expression (DGE) analysis of 56,493 genes was performed by confronting PRC1^high^ (> median) and PRC1^low^ (< median) samples from 36 cancer types (detailed information in Additional File 1). A total of 58 genes were found overexpressed in the PRC1^high^ vs. PRC1^low^ tumors in at least 18 cancer types, while 20 genes were significantly underexpressed in PRC1^high^ vs. PRC1^low^ groups (Table S2).

GSEA-based pathway analysis, including Reactome pathway, KEGG pathway, Biocarta pathway, and Wikipathway, was then performed to explore the biological function and underlying signaling pathways affected by PRC1 in the 36 cancer types (detailed enrichment in Additional File 2). Consistent with the scRNAseq results (Figures S9), cell cycle-related genes were enriched in PRC1^high^ status in all 36 cancer types (Fig. 1D). In addition, PRC1 is positively associated with DNA damage and repair in all cancer types except COAD (Fig. 1E-F). Furthermore, DEGs were enriched with respect to actin cytoskeleton, microtubule cytoskeleton, focal adhesion, cell migration, invasion, EMT process and the RHO GTPase-related pathway, in 31 out of 36 cancer types (not in CHOL, COAD, SKCM, STAD and TGCT) (Figure S10A-I). Moreover, genes involved in PI3K/AKT, ERK, NF-κB, TGF-β, RAS/RAF/MAPK, Hedgehog, Integrin, EGFR/VEGFR, JAK/STAT, MYC, WNT or NOTCH related signaling pathways, were enriched in PRC1^high^ samples from 33 out of 36 cancer types (not in CHOL, OSCC and TGCT) (Figure S11-1 A-I, Figure S11-2 A-E), supporting the positive correlation of PRC1 with proliferation.

**Fig. 2:**
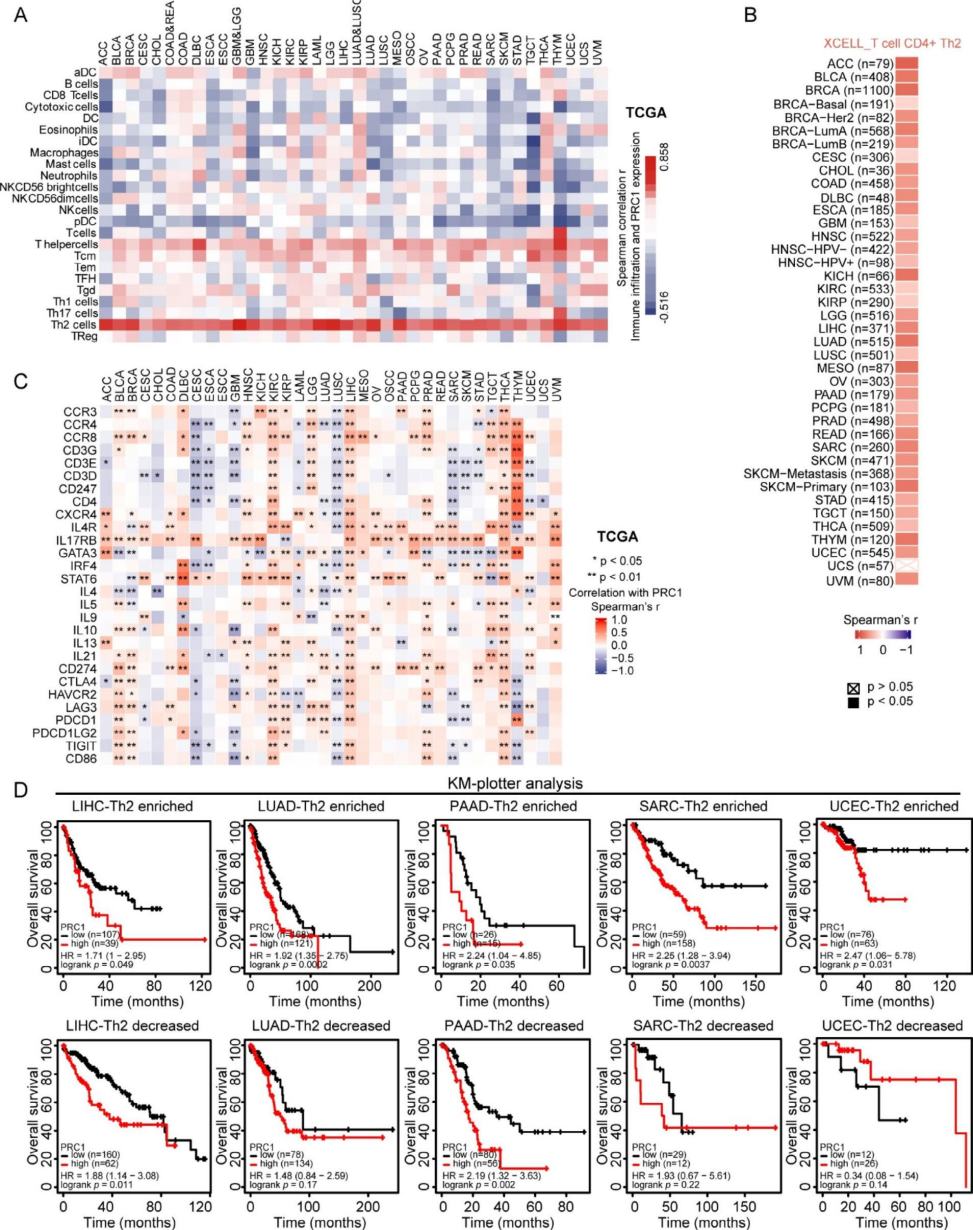
Association of PRC1 with tumor microenvironment. (**A**) Correlation of PRC1 with 24 immune cell types in 36 cancers. (**B**) Extraction of XCELL datasets indicate a significantly positive correlation of PRC1 expression and Th2 cell enrichment in these 36 cancer types. (**C**) Correlation of PRC1, Th2 cell markers and immune checkpoint genes presented as heatmaps. Statistical significance is shown as *p < 0.05, **p < 0.01, and ***p < 0.001. (**D**) Survival impact of PRC1 expression in cancers with enhanced or reduced Th2 cell level determined by means of the KM-plotter online tool

### PRC1-based gene correlation and co-expression analysis in cancer

On the basis of RNA-seq data (Log_2_ (TMP + 1)) from TCGA database, the Spearman correlations of PRC1 with 56,493 genes in the 36 cancer types were explored (detailed information in Additional File 3). In total, 163 genes were positively associated with PRC1 expression (r > 0.3 and P < 0.05) in all 36 cancer types, contrasting with the fact that not a single gene negatively correlated with PRC1 expression in all cancer types (Table S2). Combined with the 58 genes that correlated with PRC1^high^ status of at least 18 cancer types, 40 genes were defined as PRC1- co-expressed if they satisfied two conditions: log2FC > 1 & P < 0.05 and r > 0.3 & P < 0.05 (Table S2). The correlation heatmaps of PRC1 and the 40 co-expression genes in 36 cancer types are shown in Figure S12. The co-expression heatmaps of the 40 genes in 36 cancer types grouped by PRC1 expression are shown in Figure S13-(1–4), respectively.

Further analysis of protein-protein interactions (PPIs) validated the functional and physical protein associations of PRC1 and the 40 co-expressed genes, with an average local clustering coefficient of 0.944 and a PPI enrichment P value < 1.0e-16 (Figure S14A). Gene ontology (GO) and pathway analyses confirmed the involvement of PRC1 and its 40 co-expressed genes in cell cycle, DNA repair, DNA damage, RHO GTPse related pathway, microtubule cytoskeleton organization, and chromosome segregation, organization, as well as condensation (Figure S14B-C).

### Upstream regulators of PRC1

DNA methylation is an essential epigenetic factor that influences gene activities. Intriguingly, we observe that the methylation of two CpG sites, cg14398957 and cg01407062, might lead to PRC1 silencing, as the methylation level of these two sites negatively correlated with PRC1 mRNA expression in the majority of cancer types (Figure S15). More importantly, methylation of these two CpG sites indicated favorable prognostic in several cancers including ACC, KIRP, LUAD, MESO, KIRP and LGG (Fig. 1G).

The potential transcription factors (TFs) regulating PRC1 expression were predicted by 6 databases, including HumanTFDB, PROMO, CistromeDB Toolkit, hTFtarget, Genecard, and JASPAR (Additional File 4, Table S2-S3). 147 TFs were predicted by ≥ 3 databases (Fig. 12). KnockTF2.0 is a database that contains RNA-seq data and microarray datasets of knockdown/knockout of TFs by siRNA/shRNA/CRISPR in certain tissues or cell types. This database listed 21 and 67 TFs that down- and upregulate PRC1 expression, respectively (Fig. 1H, Figure S16, Additional file 4, Table S3-S4). Among these functionally relevant TFs, 24 warrant specific attention, as they are predicted by ≥ 3 databases (Fig. 12) to upregulate PRC1 mRNA (16 TFs including JUN, FOS, FLI1, MYC, MYB, STAT3, RUNX1, NFYA, ZFX, GATA1, TFAP4, NFE2L2, KLF4, FOXA1, AR, and CREB1) or downregulate PRC1 mRNA (9 TFs including JUN, REST, TP53, TFAP2C, HNF1A, ELK1, NFATC1, HSF1, and IKZF1).

Five databases (TargetScanHuman_8.0, miRWalk and miRDB, MicroT-CDS, and TarbaseV7.0) were used to explore micro RNAs (miRNAs) targeting PRC1 (for details see Additional File 5, Tables S5-S6). Sixty one miRNAs have great potential to silence PRC1, as they were predicted by ≥ 3 databases (Fig. 1I, Figure S17). The analysis of Tarbase v8.0 dataset was then conducted to identify 69 experimentally validated PRC1-targeting miRNAs (Additional File 5, Figure S17). Among these, 10 miRNAs overlapped between the correlative and functional analyses(Fig. 1I).

### PRC1-associated immune infiltration in cancer

To understand the immune-regulating roles of PRC1 in cancers, the correlation of PRC1 and immune cell infiltration of 24 cell types were explored in the TCGA. PRC1 was positively correlated with the enrichment of CD4^+^ T lymphocytes belonging to the Type 2 helper class (TH2 cells) in all the 36 cancer types (r > 0.3, P < 0.05). In contrast, PRC1 was positively or negatively associated with the infiltration of other immune cells depending on the cancer type (Fig. 2A, Figure S18, Figure S19). Consistently, the results of TIMER2.0 also suggested a positive correlation between PRC1 and TH2 enrichment in cancers (Fig. 2B). In addition, the enrichment score (ES) of TH2 cells is larger in PRC1^high^ compared to PRC1^low^ samples in 35 out of 36 cancer types (with the exception of CHOL) (Figure S20). PRC1 was significantly related with most TH2 cell markers (CCR3, CCR4, CCR8, CD3G, CD3E, CD247, CD4, CXCR4, IL4R, IL17RB, CATA3, IRF4, STAT6, IL4, IL5, IL9, IL10, IL13, and IL21) in some cancers, including KIRC, LGG, LIHC, PRAD, THCA and THYM (Fig. 2C). Furthermore, positive correlations of PRC1 and some immune checkpoint genes (CD274, CTLA4, HAVCR2, LAG3, PDCD1, PDCD1LG2, TIGIT, and CD86) were observed in several cancers, especially BLCA, BRCA, KIRC, LUAD, LIHC, PRAD and THCA (Fig. 2C). We then evaluated the prognostic value of TH2 cells based on PRC1 expression. TH2 cell enrichment predicted poor outcome of cancers including KIRP, MESO, PAAD, and KIRC, which depended on PRC1 expression (Figure S21). In addition, high PRC1 mRNA predicted poor outcome predictor in patients with high levels with intratumoral TH2 cells in LICH, LUAD, PAAD, SARC, and UCEC. This predictive value of PRC1 was lost for LUAD, SARC and UCEC tumors poor in TH2 suggesting a significant interaction between PRC1 and TH2 cell infiltration (Fig. 2D).

## Discussion

PRC1 (Protein regulator of cytokinesis 1) has been pinpointed as an unfavorable prognostic factor in several solid tumors, including breast cancer, hepatocellular carcinoma (HCC), prostate cancer (PCa), lung adenocarcinoma (LUAD), and gastric cancer [2–6]. Recently, PRC1 overexpression was further confirmed in colon cancer, ovarian cancer and Ewing sarcoma at mRNA and protein levels, and PRC1 elevation contributes to poor clinical outcome of patients with these cancers [7–9]. However, the clinical significance of PRC1 in other cancer types has remained largely unknown. Our differential *in silico* expression analysis suggested high expression of PRC1 in most solid tumors. In addition, survival analyses emphasized the great potential of PRC1 serving as an unfavorable biomarker in ACC, LGG, KICH, KIRP, LIHC, LUAD, MESO, PAAD, SARC and UCEC. To the best of our knowledge, the clinical correlation of PRC1 with ACC, LGG, KICH, KIRP, MESO, PAAD and UCEC prognosis has not not been reported thus far.

PRC1 functions as a tumor promoter in cancers [3, 5–7, 10–12]. Among all the reported cancer types, the functional roles of PRC1 has been best demonstrated in HCC. Indeed, PRC1 is tightly associated with cell proliferation, EMT (epithelial mesenchymal transition), migration, invasion, stemness, metastasis, and tumorigenesis in HCC by engaging in a positive feedback loop with Wnt/β-catenin signaling [3]. Accordingly, depletion of PRC1 attenuated cell proliferation, invasion and self-renewal, while inducing G_2_/M cell cycle arrest and apoptosis, in LUAD and esophageal cancer cells, through interruption of the Wnt/β-catenin pathway [5]. In addition, a PRC1/EGFR signaling pathway has been implicated in the growth of OSCC [10]. Similar roles of PRC1 have been confirmed in other cancers, such as liposarcoma, gastric, colon and nasopharyngeal carcinoma [6, 7, 11, 12]. Our pancancer analysis indicates that PRC1 is broadly correlated with cell cycle, proliferation, EMT, invasion, DNA damage, and DNA repair (Figure S9), which is consistent with previous investigations [3, 5–7, 10–12]. Of note, cell cycle-related genes were upregulated in PRC1^high^ tumor from all the 36 cancer types. Furthermore, PRC1 was positively associated with DNA damage and DNA repair in all cancer types except COAD. Strong positive associations were also identified between PRC1 and pathway relevant to tropic signals, as well as to cell migration and invasion in the majority of cancer types.

To date, there is evidence that transcription factor EWSR1-FLI1 can hijack PRC1 to promote tumor growth of Ewing sarcoma, while p53 directly inhibits PRC1 to regulate cytokinesis in several cancer cells lines (HCT116, MCF-7, and T47D) [3]. Furthermore, miR-194 has been demonstrated to suppress PRC1, thereby preventing tumorigenesis of esophageal cancer, inhibiting cell migration and invasion in HCC, and affecting radiosensitivity and metastasis of tumor cells in nasopharyngeal carcinoma [5]. In addition to miR-194, PRC1 is also a target of miR-143 (liposarcoma) and miR-1-3p (LUAD), which exhibit antiproliferative effects [6, 10, 11]. Here, we identified additional transcription factors and miRNAs that affect PRC1 mRNA expression. Furthermore, the methylation of 2 CpG sites, cg14398957 and cg01407062, negatively correlated with PRC1 expression, and this methylation had favorable prognostic value for several cancers (Fig. 2I, Figure S21).

Tumor development and progression are dynamic and complex processes, which are reliant on the crosstalk between tumor and its immune microenvironment. Notably, we found that PRC1 correlates with the enrichment of TH2 cells in all 36 cancer types. Indeed, TH2 cells are closely associated with chronic inflammation, which is believed tumor promoting factor [15]. Interestingly, further survival analyses indicate that the unfavorable prognostic value of PRC1 is TH2 cell-dependent in LUAD, SARC, and UCEC. It is also noteworthy that PRC1 also correlates positively with most immune checkpoint genes (CD274, CTLA4, HAVCR2, LAG3, PDCD1, PDCD1LG2, TIGIT, and CD86) in KIRC, LIHC, PRAD, and THCA, implying that PRC1 might contribute to an immunosuppressive microenvironment. Immunohistochemical and proteomic studies measuring the abundance of PRC1 protein (instead of mRNA) in multiple cancer types are needed to substantiate the hypothesis that PRC1 expression has a prognostic and immunological impact on tumor progression.

Thus far there are no therapeutic agents directly targeting PRC1. PLK1 and CDK16 function by phosphorylating PRC1 [9], meaning that CDK16 and PLK1 inhibitors exert indirect inhibitory effect on PRC1. Intriguingly, 88 chemicals decrease the expression of PRC1 at mRNA/protein levels or decreases phosphorylation of PRC1 protein in *Homo sapiens*, *Mus musculus*, or *Rattus norvegicus* based on the information from the Comparative Toxicogenomics Database (Additional File 6). However, such drugs have not been evaluated yet for their potential anticancer effects. It would be particularly interesting to test such putative PRC1 inhibitors for their effects on the immunosuppressive TH2-driven microenvironment, alone or in combination with currently used immune checkpoint inhibitors.

## Conclusion

PRC1 is highly overexpressed in the vast majority of cancers, and this overabundance has a poor prognostic impact especially in ACC, LGG, KIRP, LICH, LUAD, MESO, PAAD, SARC and UCEC. Correlative evidence suggests PRC1 to be involved in key signaling pathways associated with cell cycle, DNA damage, DNA repair, EMT, migration, invasion and proliferation. The upstream mechanisms responsible for regulating PRC1 expression involve various TFs, miRNAs and DNA methylation. The correlation of PRC1 with a TH2-rich immunosuppressive tumor microenvironment may encourage future attempts to target PRC1 in the context of immunotherapies.

### Electronic supplementary material

Below is the link to the electronic supplementary material.

**Supplemental text file 1: Materials and Methods**.
**Additional file 1**: Differentially expressed genes (56493 genes) in tumor tissues compared by PRC1: High vs. Low in 36 cancer types, the data is separately shown in sheets 1–36 in 36 cancer types; **Additional file 2.** GSEA-based pathway analysis of the differentially expressed genes (56493 genes) in 36 cancers, the data is separately shown in sheets 1–36 in 36 cancer types; **Additional file 3.** The correlation analysis of PRC1 with the 56493 genes in 36 cancers, the data is separately shown in sheets 1–36 in 36 cancer types; **Additional file 4.** The predicted and experimentally validated transcription factors upstream of PRC1, **Additional file 5.** The predicted and validated miRNAs targeting PRC1, and **Additional file 6.** Chemicals that affect the expression/phosphorylation of PRC1.

## Data Availability

All data generated or analyzed during this study are included in this published article and its supplementary information files.

## Abbreviations

PRC1: Protein regulator of cytokinesis 1
PCa: Prostate cancer
ACC: Adrenocortical carcinoma
BLCA: Bladder Urothelial Carcinoma
BRCA: Breast invasive carcinoma
CESC: Cervical squamous cell carcinoma and endocervical adenocarcinoma
CHOL: Cholangio carcinoma
COAD: Colon adenocarcinoma
DLBC: Lymphoid Neoplasm Diffuse Large B-cell Lymphoma
ESAD: Esophageal Adenocarcinoma
ESCA: Esophageal carcinoma
ESCC: Esophageal squamous-cell carcinomas
GBM: Glioblastoma multiforme
HNSC: Head and Neck squamous cell carcinoma
KICH: Kidney Chromophobe
KIRC: Kidney renal clear cell carcinoma
KIRP: Kidney renal papillary cell carcinoma
LAML: Acute Myeloid Leukemia
LGG: Brain Lower Grade Glioma
LIHC: Liver hepatocellular carcinoma
LUAD: Lung adenocarcinoma
LUSC: Lung squamous cell carcinoma
MESO: Mesothelioma
OSCC: Oral squamous cell carcinoma
OV: Ovarian serous cystadenocarcinoma
PAAD: Pancreatic adenocarcinoma
PCPG: Pheochromocytoma and Paraganglioma
PRAD: Prostate adenocarcinoma
READ: Rectum adenocarcinoma
SARC: Sarcoma
SKCM: Skin Cutaneous Melanoma
STAD: Stomach adenocarcinoma
TGCT: Testicular Germ Cell Tumors
THCA: Thyroid carcinoma
THYM: Thymoma
UCEC: Uterine Corpus Endometrial Carcinoma
UCS: Uterine Carcinosarcoma
UVM: Uveal Melanoma
DSS: Disease Specific Survival
OS: Overall Survival
PFI: Progression Free Interval
RFS: Relapse Free Survival
EMT: Eepithelial–Mesenchymal Transition
GSEA: Gene Set Enrichment Analysis
NES: Normalized enrichment score
WP: Wikipathway
PPIs: Protein–protein interactions
TFs: Transcription factors
miRNAs: microRNAs
